# Tuberculosis Infection in Chinese Patients with Giant Cell Arteritis

**DOI:** 10.1038/s41598-019-50892-9

**Published:** 2019-10-07

**Authors:** Yun Zhang, Dongmei Wang, Yue Yin, Yu Wang, Hongwei Fan, Wen Zhang, Xuejun Zeng

**Affiliations:** 1 Department of General Internal Medicine, Peking Union Medical College Hospital (PUMCH), Chinese Academy of Medical Science (CAMS) and Peking Union Medical College (PUMC), Beijing, 100730 China; 2 Department of Neurology, Nanfang Hospital, Southern Medical University, Guangzhou, 510515 China; 3 Department of Infectious Disease of PUMCH, CAMS & PUMC, Beijing, 100730 China; 4 Department of Rheumatology of PUMCH, CAMS & PUMC, Beijing, 100730 China

**Keywords:** Immunology, Rheumatology

## Abstract

Giant cell arteritis (GCA) is a medium- and large-vessel vasculitis with an onset age after 50 years. Takayasu arteritis (TA), which is also a large-vessel vasculitis with an onset age earlier than 40 years, was suggested to be associated with tuberculosis (TB). However, the association between GCA and TB was rarely reported. This study was to retrospectively analyze clinical data of GCA patients at Peking Union Medical College Hospital and elucidate the association between GCA and TB. Ninety-one patients diagnosed with GCA were included in the study. A total of 20 patients (22.0%) had a history of active tuberculosis and received anti-tuberculosis therapy. On comparing the clinical features of patients with GCA and concomitant TB and those without TB, obvious weight loss (*P* = 0.011), lower percentage of dyslipidemia (*P* = 0.042), higher percentage of anti-phospholipid antibodies (*P* = 0.010), and lower white blood cells (*P* = 0.006) were noted in the TB group. In conclusion, this study demonstrated the percentage of TB history in patients with GCA was higher than that in the Chinese general population. Clinicians should recognize the possibility of comorbid TB in patients with obvious weight loss and relatively lower white blood cell count.

## Introduction

Giant cell arteritis (GCA) is a medium- and large-vessel vasculitis, which is an important cause of secondary headache in older adults^1^. It has a predilection for the branches of the carotid and vertebral arteries, and an onset usually after the age of 50^2^. The GCA is diagnosed according to the 1990 American College of Rheumatology criteria, which requires fulfillment of three out of five core features. These include age of 50 years or older at onset, a new headache, a clinical temporal artery abnormality, an elevated erythrocyte sedimentation rate (ESR) of at least 50 mm/h, and an abnormal temporal artery biopsy^3^.

As we know, large-vessel vasculitis consists of GCA and Takayasu arteritis (TA), and the onset age of the latter one is usually before 40^4^. An association between TA and infection with *Mycobacterium tuberculosis* has been suggested due to the high prevalence of TA in countries with a high prevalence of TB and the granulomatous nature of both diseases^4,5^. However, the association between GCA and tuberculosis (TB) is rarely reported^6^. Diagnosing GCA and TB, especially among the elderly, is a challenge because both diseases have similar clinical symptoms, including headache, visual and constitutional symptoms, and elevated acute reactive proteins. TB diagnosis is extremely difficult because of its broad, nonspecific clinical manifestations, accounting for 12%–20% of cases of fever of unknown origin (FUO) in the elderly^6,7^. GCA can also present as FUO. Therefore, it is essential to identify whether it is isolated GCA or TB or both.

The association between TA and TB is widely suggested, while little is known about the association between GCA and TB. This study aimed to retrospectively analyze patients with GCA and elucidate the association between GCA and TB. Also, the clinical characteristics of patients with GCA with and without concomitant tuberculosis were compared.

## Results

### Study population

Table 1 illustrates the sociodemographic data of the study population. Of the 91 patients, 50 were female (54.9%), and the male-to-female ratio was 0.82:1. Twenty patients (22.0%) were diagnosed with comorbidities of TB. The mean age at the diagnosis of symptoms was 65.10 ± 8.39 years old in patients with GCA with TB and 65.38 ± 7.51 years old in patients with GCA without TB (P = 0.886). The disease duration from onset to diagnosis was 5.35 ± 5.59 months in patients with GCA with TB and 9.71 ± 19.31 months in patients with GCA without TB (P = 0.322). Both groups had similar delayed time course from the onset of symptoms to the time of diagnosis. The proportion of female patients was not significantly different between the two groups (P = 0.126).

**Table 1:** Clinical features and comorbid diseases of the patients with TB and without TB.

	GCA with TB *n* = 20 (%)	GCA without TB *n* = 71 (%)	*P* value
**Sociodemography**			
Age (years, diagnosis)	65.10 ± 8.39	65.38 ± 7.51	0.886
Disease course (months)	5.35 ± 5.59	9.71 ± 19.31	0.322
Sex			
Male	6 (30)	35 (49.3)	0.126
Female	14 (70)	36 (50.7)	
**Clinical signs and symptoms**			
Headache	6 (30)	21 (29.6)	0.971
Fever	16 (80)	58 (81.7)	0.864
Scalp tenderness or pain	2 (10)	22 (31.0)	0.060
Tenderness and abnormal pulsation of temporal artery	6 (30)	13 (18.3)	0.256
Visual loss	8 (40)	25 (35.2)	0.694
Myalgia	12 (60)	39 (54.9)	0.687
CNS symptoms	(25)	16 (22.5)	0.817
Hearing loss	3 (15)	20 (28.2)	0.231
Jaw claudication	6 (30)	20 (28.2)	0.873
Arthralgia	11 (55)	36 (50.7)	0.734
GI symptoms	1 (5)	11 (15.5)	0.221
Constitutional symptoms	14 (70)	47 (66.2)	0.749
Weight loss^*^	16 (80)	34 (47.9)	**0.011**
**Comorbid diseases**			
Atherosclerosis	4 (20)	28 (39.4)	0.108
Smoking	6 (30)	27 (38.0)	0.509
Diabetes	1 (5)	13 (18.3)	0.145
Cerebrovascular disease	2 (10)	6 (8.5)	0.829
Coronary artery disease	2 (10)	11 (15.5)	0.535
Hypertension	9 (45)	23 (32.4)	0.297
Dyslipidemia^*^	3 (15)	28 (39.4)	**0.042**

CNS symptoms: vertigo, transient ischemia attack, and stroke; GI in volvement: abdominal pain and abdominal distention; constitutional symptom: fatigue, night sweat, and anorexia.

CNS, Central nervous system; GCA, giant cell arteritis; GI, gastrointestinal; TB, tuberculosis.

*Significantly different.

Of the 20 patients categorized into the group with TB, 16 had pulmonary TB, one had TB pleuritis, one had pulmonary TB comorbid with TB pleuritis, one had TB lymphadenitis of the mesentery, and the last one had pulmonary TB, TB of sternum, and TB peritonitis.

One of them was diagnosed with open pulmonary tuberculosis with positive M. tuberculosis in the sputum. Five patients were highly suspected with potentially active tuberculosis. Anti-TB therapy was administered using steroids and immunosuppressant medications. During the follow-up, one patient with old pulmonary tuberculosis was diagnosed with TB pleuritis and anti-TB therapy was initiated. During the follow-up, 3 patients were lost to follow up in the GCA with TB and 11 in GCA without TB (Table 2). Patients were categorized into four subsets according to their symptoms, including non-relapsing GCA, relapsing GCA, comorbid with malignant tumors, and death. The proportion of GCA-relapsing subset was significantly lower in the GCA with TB group than in the GCA without TB group (P = 0.027). No significant differences were observed in other three subsets.

**Table 2:** Laboratory findings and follow-up results of GCA patients according to TB.

	GCA with TB			GCA without TB			*P* value
	Mean ± SD (range)	*n* (%)	Total *n*	Mean ± SD (range)	*n* (%)	Total *n*	
**Laboratory findings**							
ESR (mm/h)	94.50 ± 28.21 (44−140)		20	90.01 ± 27.74 (21−140)		71	0.526
CRP (mg/L)	76.71 ± 59.67 (2.47−197)		20	80.68 ± 63.45 (1.8−275.66)		71	0.803
ALB (g/L)	33.20 ± 5.65 (24−47)		20	32.38 ± 4.30 (22−42)		69	0.486
WBC ( × 10^9^/L) ^*^	6.92 ± 2.39 (1.47−10.91)		20	9.80 ± 4.42 (2.36−26.60)		69	**0.006**
Leukopenia		3 (15)	20		3 (4.3)	69	0.148
LYM ( × 10^9^/L)	1.65 ± 0.67 (0.62−2.94)		20	1.63 ± 0.78 (0.40−4.20)		69	0.914
HGB (g/L)	100.30 ± 17.14 (63−132)		20	108.84 ± 19.86 (63−146)		69	0.085
PLT ( × 10^9^/L)	400.20 ± 151.07 (130−653)		20	373.75 ± 152.83 (99−725)		69	0.496
Fbg (g/L)	6.25 ± 2.69 (2.90−10.00)		20	5.30 ± 2.12 (2.00−10.49)		57	0.100
ANA positive		6 (30)	20		12 (17.6)	68	0.229
ANCA positive		1 (5)	20		12 (18.8)	64	0.138
APS positive^*^		5 (55.6)	9		5 (14.7)	34	**0.010**
		4 LA + 1 ACL+			4 ACL + 1 β 2GP1 + 1 positive for 3 antibodies		
Elevated RF		2 (15.4)	13		17 (33.3)	51	0.206
Elevated IgG^*^		7 (50)	14		14 (23.3)	60	**0.046**
**Follow-up results**							
Non-relapsing		11 (64.7)	17		26 (43.3)	60	0.119
Relapsing		2 (11.8)	17		24 (40)	60	**0.027**
Comorbid with tumors		1 (5.9)	17		1 (1.7)	60	0.335
Death		3 (17.6)	17		9 (15)	60	0.791

ALB, Albumin; ACL, anti-cardiolipin antibody; ANA, anti-nuclear antibody; ANCA, anti-neutrophil cytoplasmic antibody; APS, anti-phospholipid antibody; β 2GP1, β2glycoprotein1; CRP, C-reactive protein; ESR, erythrocyte sedition rate; Fbg, fibrinogen; GCA, giant cell arteritis HGB, hemoglobin; Ig G, immunoglobin G; LA, lupus anticoagulant; LYM, lymphocyte; PLT, platelet; RF, rheumatic factor; SD, standard deviation; TB, tuberculosis; WBC, white blood cell. ^*^Significantly different.

### Comparisons of clinical manifestations

Clinical features and comorbid diseases of the patients were taken from medical records at GCA presentation and they are described in Table 1. Clinical features, including headache, fever, scalp tenderness or pain, tenderness and abnormal pulsation of temporal artery, visual loss, myalgia, central nervous system symptoms (vertigo, transient ischemia attack, and stroke), hearing loss, jaw claudication, arthralgia, gastrointestinal symptoms (abdominal pain and abdominal distention), and constitutional symptoms (fatigue, night sweat, and anorexia) were not significantly different between patients with GCA with TB and GCA without TB. Weight loss was significantly reported in patients with GCA with TB (P = 0.011). Regarding the comorbid diseases, including arteriosclerosis, smoking, diabetes, coronary artery disease, cerebrovascular disease, and hypertension were not significantly different between patients with GCA with TB or GCA without TB. Dyslipidemia was highly reported in patients without TB (P = 0.042).

Laboratory results, including ESR, C-reactive protein (CRP), albumin, hemoglobin (HGB), white blood cell (WBC) count, platelet (PLT) count, fibrinogen (Fbg), anti-nuclear antibody (ANA), anti-neutrophil cytoplasmic antibody (ANCA), anti-phospholipid antibody (APS, lupus anticoagulant, anti-cardiolipin antibody, β-2-glycoprotein 1), immunoglobulin G (IgG), and rheumatic factor (RF), were evaluated (Table 2). The WBC count was significantly lower in patients with TB compared with patients without TB (P = 0.006). The frequency of positive APS and elevated IgG was significantly higher in patients with TB. ESR and CRP were not significantly different between the two groups. The immunological antibodies, including ANA, ANCA, APS, and RF, were not significantly different between the two groups.

## Discussion

This novel study retrospectively reviewed the data of GCA and found an association between TB and GCA.

This study demonstrated that TB infection history accounted for 22.0% of patients with GCA. Based on the survey performed by the China Center for Disease Control, the prevalence of smear-positive tuberculosis decreased from 170 cases (95% CI 166–174) to 59 cases (49–72) per 100,000 population between 1990 and 2010^8^. A study performed to explore the prevalence of latent TB infections in China demonstrated that skin-test positivity (≥10 mm) ranged from 15% to 42%, and interferon-γ release assay [QuantiFERON (QFT)] positivity rates ranged from 13% to 20%^9^. It is estimated that 5–10% of patients with latent tuberculosis will develop active disease^9^. Based on these data, the percentage of patients with active TB infection was 0.75–4%, although the proportion of TB infection history of Chinese population was not reported. The occurrence of TB in systemic lupus erythematosus (SLE) was 3.3–9.29%, which was significantly higher than that in the general population in China^10,11^. In this study, the occurrence of TB history in GCA was 22.0%, which was much higher than that in patients with SLE and also the general population.

The association between TB and TA was widely suggested^5,12^. TB is indicated as a trigger of TA. The suggested mechanism was cross-reactivity of the immune response between mycobacterial heat shock protein and a human homolog^13^. Similar phenomena were also observed in other autoimmune-mediated diseases^14,15^. The association between TB and GCA was not widely reported, although several case reports suggested the potential correlation between the two diseases^6^. The present study precisely evaluated the TB history of patients and found that the proportion of TB history was fourfold higher than that in the general population. This finding strongly suggested the association between TB and GCA. GCA patients in Chinese population might need to be screened for TB before the institution of immunosuppressive drugs and GCA patient with TB in China need to be paid attention to the recurrence of TB while using immunosuppressive drugs. However, the underlying mechanisms for this association remained unknown. Based on the robust research data of TB and autoimmune diseases, cross-reactivity of the immune response and adjuvant effect of the mycobacterial component were suggested to be involved in the pathogenesis of GCA^14^. However, more studies are required to elucidate the roles of TB in patients with GCA.

GCA and TB share clinical features and radiological features, especially in elderly patients. Clinicians should differentiate between the two diseases carefully because the treatment is quite different. In the present study, five patients were treated with anti-TB therapy when steroids and immunosuppressant were prescribed for GCA. During the follow-up, one patient was diagnosed with TB pleuritis, and tapering of steroids and immunosuppressants was required. Clinicians should monitor patients with TB history carefully to avoid recurrence of TB infections.

With respect to the clinical features and comorbid diseases, patients with TB were reported to have significant weight loss and a lower rate of dyslipidemia compared with patients without TB. This finding was consistent with the common clinical features of TB infection, including weight loss, low-grade fever, and night sweats. Other clinical features had no significant differences between the two groups. Therefore, TB should be considered in patients with GCA having obvious weight loss.

The laboratory results showed that WBC count was significantly lower in patients with TB compared with patients without TB. The frequency of leukopenia was not significantly different between the two groups. A relatively lower WBC count was considered to be a common feature in patients with TB. Leukopenia in TB infection was widely reported^16,17^. A recent study of 1440 newly treated patients with TB and 464 previously treated patients with TB demonstrated that the prevalence of leukopenia in newly treated patients was 150 (10.4%), while the prevalence in previously treated patients was 42 (9.1%)^17^. Anemia was also found in both groups in this study. Although the hemoglobin (Hb) levels of the two groups were not significantly different, the average Hb level in the TB group was lower. According to the published studies, anemia was a common feature in patients with TB, and in severe cases, especially military TB, pancytopenia, even hemophagocytic lymphohistiocytosis, could occur^18,19^.

Another interesting finding was that the frequency of positive APS antibody was significantly higher in the TB group, although the sample size was really small. APS antibodies increased in chronic TB infection in both animal models and humans^20,21^. APS was considered to be a biomarker to monitor the efficacy of anti-TB treatment^22^.

With respect to the follow-up results, the percentage of unstable patients was significantly lower in the GCA with TB group. This phenomenon might be due to the association between GCA and TB, which remains unknown. This result might be owing to the bias caused by a small sample size.

This study had some limitations. It was a single-center study with a limited sample size. Also, it retrospectively analyzed data collected from patients with obvious patient loss during the follow-up.

In conclusion, this study retrospectively analyzed patients with GCA. It found that 22% of these patients had a definite history of TB, which was obviously higher than the percentage of TB history in the general population. On comparing the patients with TB and those without TB, the clinical features and laboratory results showed that GCA with TB had a higher percentage of weight loss and a lower frequency of dyslipidemia. Moreover, a lower WBC count and a higher frequency of anti-phospholipid antibodies were noted. TB screening is suggested once the patients demonstrate apparent weight loss and leukopenia. Although the present study reported a high percentage of TB history, indicating potential role of TB in the pathogenesis of GCA, more studies are required to elucidate the relationship between GCA and TB.

## Methods

### Patients

Ninety-one patients diagnosed with GCA between November 1998 and October 2017 at Peking Union Medical College Hospital, Beijing, China, were identified. All patients fulfilled the 1990 American College of Rheumatology diagnostic criteria for GCA^3^. Fifty-five of the patients underwent temporal artery biopsy, and 46 patients were pathologically diagnosed with GCA.

All the patients were followed up for evaluating clinical symptoms of tuberculosis, including low-grade fever, fatigue, night sweat, cough, and weight loss. Their median follow-up period was 86.04 (3–218) months.

### Clinical data collection

Clinical characteristics, including signs and symptoms, laboratory findings, and angiographic findings at the time of diagnosis, from the medical records of patients with GCA, were retrospectively reviewed. The comorbid diseases included arteriosclerosis, smoking, diabetes, coronary artery disease, cerebrovascular disease, hypertension, and dyslipidemia. TB infection history was identified based on previously diagnosed TB with long-time regular anti-TB therapy. Current TB infection was comprehensively evaluated based on clinical signs, symptoms, and clinical tests, including T-SPOT.TB test (enzyme-linked immunospot assay for T-cell reaction to M. tuberculosis–specific antigens), chest x-ray or computed tomography (CT), and sputum pathogen tests.

For vascular evaluation, radiologic data, including digital subtraction angiography, ultrasonography, CT angiography, MR angiography, and positron emission tomography, were reviewed. Forty-six patients were diagnosed with temporal artery biopsy, and pathology data were also reviewed to reconfirm the diagnosis.

Patients were followed up and evaluated. Patients who underwent therapy and recovered gradually without recurrence were considered stable. Patients who underwent therapy and had recurrence of GCA or comorbid infections were considered unstable.

### Statistical analysis

The data were analyzed using the SPSS 20.0 software (SPSS, IL, USA). The normally distributed continuous variables were summarized using mean and standard deviation, non-normally distributed variables using median, and categorical variables using absolute frequencies and percentages. Chi-square tests were used to compare categorical variables, and Student t tests were used to compare continuous variables between the studied groups. All statistical tests were two sided, and P values less than 0.05 were considered to be statistically significant.

### Ethical approval and informed consent

This study has been approved by the Ethics Board of Peking Union Medical College Hospital, and the ethics approval number is S-K437.

All procedures and methods followed were in accordance with the ethical standards of the responsible committee on human experimentation (institutional and national) and with the Helsinki Declaration of 1975, as revised in 2000.

Informed consent was obtained from all patients included in the study.

## Data Availability

All the authors promise to make materials, data and associated protocols promptly available to readers without undue qualifications in material transfer agreements.
